# Adaptation to low pH and lignocellulosic inhibitors resulting in ethanolic fermentation and growth of *Saccharomyces cerevisiae*

**DOI:** 10.1186/s13568-016-0234-8

**Published:** 2016-08-26

**Authors:** Venkatachalam Narayanan, Violeta Sànchez i Nogué, Ed W. J. van Niel, Marie F. Gorwa-Grauslund

**Affiliations:** 1 Division of Applied Microbiology, Department of Chemistry, Lund University, P.O. Box 124, 221 00 Lund, Sweden; 2 National Bioenergy Center, National Renewable Energy Laboratory, 15013 Denver West Parkway, Golden, CO 80401 USA

**Keywords:** *Saccharomyces cerevisiae*, Low pH, Lignocellulosic inhibitors, Phenotypic robustness, Adaptation, Ethanol yield

## Abstract

Lignocellulosic bioethanol from renewable feedstocks using *Saccharomyces cerevisiae* is a promising alternative to fossil fuels owing to environmental challenges. *S. cerevisiae* is frequently challenged by bacterial contamination and a combination of lignocellulosic inhibitors formed during the pre-treatment, in terms of growth, ethanol yield and productivity. We investigated the phenotypic robustness of a brewing yeast strain TMB3500 and its ability to adapt to low pH thereby preventing bacterial contamination along with lignocellulosic inhibitors by short-term adaptation and adaptive lab evolution (ALE). The short-term adaptation strategy was used to investigate the inherent ability of strain TMB3500 to activate a robust phenotype involving pre-culturing yeast cells in defined medium with lignocellulosic inhibitors at pH 5.0 until late exponential phase prior to inoculating them in defined media with the same inhibitor cocktail at pH 3.7. Adapted cells were able to grow aerobically, ferment anaerobically (glucose exhaustion by 19 ± 5 h to yield 0.45 ± 0.01 g ethanol g glucose^−1^) and portray significant detoxification of inhibitors at pH 3.7, when compared to non-adapted cells. ALE was performed to investigate whether a stable strain could be developed to grow and ferment at low pH with lignocellulosic inhibitors in a continuous suspension culture. Though a robust population was obtained after 3600 h with an ability to grow and ferment at pH 3.7 with inhibitors, inhibitor robustness was not stable as indicated by the characterisation of the evolved culture possibly due to phenotypic plasticity. With further research, this short-term adaptation and low pH strategy could be successfully applied in lignocellulosic ethanol plants to prevent bacterial contamination.

**Electronic supplementary material:**

The online version of this article (doi:10.1186/s13568-016-0234-8) contains supplementary material, which is available to authorized users.

## Introduction

Increasing concerns over the need for sustainable and scalable fuels as a means to curb global warming has led to focus on bioethanol production from renewable biomass, such as agricultural and industrial residues (Limayem and Ricke 2012). Due to the decrease in costs of petroleum as a response to recent discoveries of fossil fuel reserves, there is an intense emphasis on lowering the costs of renewable bioethanol by overcoming challenges connected to high substrate costs, low titers and low production rates accompanied by low yields (Papoutsakis and Pronk 2015).

A significant challenge in the fuel ethanol production is acute and chronic bacterial contamination, since the incoming substrate might contain microorganisms and the fermentation is carried out in non-aseptic conditions (Skinner and Leathers 2004). Bacterial contamination is predominantly due to lactic and acetic acid bacteria leading to loss of fermentable sugars and micronutrients, increased by-product formation (lactic acid and acetic acid), reduced ethanol yields and productivities and stuck fermentations (Beckner et al. 2011; Bischoff et al. 2009). Bacterial contamination has been studied extensively (Bischoff et al. 2009; Skinner and Leathers 2004) and several antimicrobial strategies, including usage of antibiotics, have been adopted in the first generation bioethanol production (Muthaiyan et al. 2011). These methods are expensive and some are environmentally invasive when used in large-scale fermentations (Muthaiyan et al. 2011). Contamination might pose a bigger threat to lignocellulosic ethanol owing to the versatility in sugar substrates. As *Saccharomyces cerevisiae* displays glucose repression, it takes longer time to assimilate other sugars including xylose and arabinose. Thus any contamination might be able to utilise the other sugars swiftly and more efficiently than *S. cerevisiae* leading to reduction in ethanol production. Several attempts have been made to study and control bacterial contamination in lignocellulosic ethanol production including: (1) adding NaCl and ethanol to wood hydrolysate (Albers et al. 2011), (2) high solid loading in simultaneous saccharification and fermentation (SSF) (Ishola et al. 2013), (3) usage of an antibiotic like gentamicin and biomass autoclaving (Serate et al. 2015), and (4) usage of bacteriophages (Worley-Morse et al. 2015). These strategies encounter challenges including: (1) additional cost and need for extensive fine tuning and testing of concentrations of NaCl and ethanol (Albers et al. 2011), (2) loss of cell viability due to mechanical stress caused by solid particles in high cell loading (Ishola et al. 2013), (3) cost and environmental challenges posed by gentamicin, energy expenditure and formation of inhibitors due to autoclaving (Serate et al. 2015), and (4) rise of bacteriophage-insensitive mutants and possibilities of gene transfer from bacteriophages to yeast (Worley-Morse et al. 2015). One of the potentially scalable and economically feasible solutions to control bacterial contamination is to run the lignocellulosic fermentation at low pH, around pH 4 where the growth and viability of bacteria are drastically reduced (Kádár et al. 2007). Additionally, yeast cells are recycled in several commercial ethanol production processes up to 6 months to reduce fermentation time and cost of yeast propagation, increasing the chances of contamination (Basso et al. 2011). To prevent contamination, yeast cells are treated with dilute sulphuric acid (H_2_SO_4_) at pH between pH 1.8 and 2.5 for 1–2 h (Basso et al. 2011), which results in reduction in intracellular pH (Beales 2004), yeast viability and low ethanol yield (De Melo et al. 2010). Hence, it might be efficient to develop *S. cerevisiae* strains tolerant to lower pH induced by inorganic acids.

Apart from bacterial contamination, inhibitors pose another obstacle to yeast in ethanol production, formed from the components of lignocellulose including cellulose, hemicellulose and lignin due to the harsh conditions of biomass pre-treatment (Almeida et al. 2007). They include (1) weak organic acids such as acetic acid, formic acid and levulinic acid, (2) furans, including furfural and hydroxymethylfurfural (HMF), and (3) phenolic compounds such as vanillin, coniferyl aldehyde and 4-hydroxybenzoic acid (Palmqvist and Hahn-Hägerdal 2000; Taherzadeh and Karimi 2011). *S. cerevisiae* endures inhibitors through different mechanisms, including detoxification by enzymatic reduction, efflux and membrane repair (Piotrowski et al. 2014). Advancements in pre-treatment processes resulted in reduction of furans, phenolics, formic acid and levulinic acid concentrations in the hydrolysate (Jönsson et al. 2013). However, acetic acid is naturally bound to lignocellulose in the form of acetyl sugars in the hemicellulose fraction and becomes de-acetylated during the hydrolysis treatment (Almeida et al. 2007). As a weak organic acid, its effect is more pronounced at low pH and may facilitate synergy between furans and phenolics (Ding et al. 2011). Hence, it is crucial to focus on yeast tolerance in acidic environments inflicted by the combination of inorganic and weak organic acids in the presence of lignocellulosic inhibitors for cost competitive ethanol production.

Different rational engineering strategies have been pursued with *S. cerevisiae* to understand the molecular mechanisms involved in coping with one or several inhibitors, thereby creating inhibitor tolerant *S. cerevisiae* strains (Alriksson et al. 2010; Caspeta et al. 2015; Koichi et al. 2012; Lei et al. 2011; Liu 2011; Parawira and Tekere 2011; Taherzadeh and Karimi 2011; Takuya et al. 2013). As Meijnen et al. (2016) found that tolerance towards acetic acid is a result of a polygenic response from yeast, evolutionary adaptation might be a suitable strategy to improve tolerance towards low pH and acetic acid with other lignocellulosic inhibitors since yeast might accrue beneficial properties under stress conditions over the time of evolution. Evolutionary engineering strategies have been successfully pursued to obtain yeast strains with enhanced tolerance against individual or combinations of several inhibitors in defined media (Dominik and Uwe 2008; Wright et al. 2011) or in hydrolysates (Almario et al. 2013; Hanqi et al. 2014) with beneficial properties including better growth, improved viability, higher yield and ethanol productivity in comparison to the control strains.

Yeast tolerance towards inhibitors could also be induced by pre-cultivation with lower concentration of inhibitors in defined media or diluted hydrolysates. This induces the general stress response leading to improved growth and fermentation performance in the inhibitory medium or hydrolysate (Nielsen et al. 2015; Tomás-Pejó and Olsson 2015). Kádár et al. (2007) had improved the tolerance of *S. cerevisiae* towards lignocellulosic inhibitors at pH 4, but with lower concentration of inhibitors to potentially threaten yeast growth and fermentation. Lowering the culture pH to pH 4 in the presence of different concentrations of acetic acid in corn-mash has resulted in complete inhibition of ethanol production at acetic acid concentrations greater than 0.8 % weight/volume (w/v) (Graves et al. 2006). Yet, other industrial strains have been analysed for their low pH tolerance, such as JP1 and PE-2 from commercial ethanol production in Brazil (De Melo et al. 2010; Della-Bianca et al. 2014). We have previously demonstrated a strain-independent pre-cultivation strategy where *S. cerevisiae* cells can grow and ferment at pH 3.7 with lethal concentrations of 6 g L^−1^ acetic acid after a short-term adaptation with 6 g L^−1^ acetic acid at pH 5.0 (Sànchez i Nogué et al. 2013).

Though numerous endeavours have been pursued to develop a robust *S. cerevisiae* strain in the presence of inhibitors by targeted, evolutionary and pre-cultivation approaches, to our knowledge, there has been no investigation on improving phenotypic robustness of *S. cerevisiae* to low pH with acetic acid and other lignocellulosic inhibitors. Herein, we aimed at developing a short-term adaptation strategy and an ALE of yeast in a chemostat to investigate the nature of adaptability of *S. cerevisiae* to the harsh conditions of low pH and lignocellulosic hydrolysates and the stability of the acquired robustness. Furthermore, understanding the interactive effects among the inhibitors at low pH could pave the way for developing new strains and strategies to lignocellulosic ethanol production.

## Materials and methods

### Yeast strain and media

The commercial *S. cerevisiae* brewer’s strain, Coobra 6 Magnum (CBF Drinkit AB, Mölndal, Sweden) was renamed to TMB3500 (Almeida et al. 2009a) and used in this study. It was stored at −80 °C in yeast peptone dextrose (YPD) medium containing 10 g L^−1^ yeast extract, 20 g L^−1^ peptone and 20 g L^−1^ glucose supplemented with 30 % (v/v) glycerol and maintained on YPD medium with 20 g L^−1^ agar. All the chemicals were purchased from Sigma Aldrich, Sweden, unless mentioned otherwise.

A chemically defined medium (Verduyn et al. 1992) with 20 g L^−1^ glucose, buffered with 50 mM potassium hydrogen phthalate and 20 mM potassium hydroxide (KOH) (Hahn-Hägerdal et al. 2005) was used in all aerobic growth experiments. The pH of the defined medium was adjusted using 3 M H_2_SO_4_ and 3 M KOH. Ergosterol and Tween80 were added in anaerobic experiments, in the final concentrations of 0.01 and 0.42 g L^−1^, respectively. A silicone based antifoam (0.5 mL L^−1^) was added in the experiments performed in fermentors to avoid excessive foaming (Dow Corning Antifoam RD emulsion, VWR International Ltd., Poole, UK). Compounds including 6 g L^−1^ acetic acid, 1.5 g L^−1^ furfural, 0.5 g L^−1^ HMF and 1 g L^−1^ vanillin were used as inhibitors in this study, hereafter mentioned as inhibitor cocktail (IC), unless mentioned otherwise. The concentrations of different inhibitors chosen in this study were in the ranges found in different pre-treated lignocellulose hydrolysates obtained from barley straw, dilute spruce and wheat straw (Almeida et al. 2009a). The used vanillin concentration of 1 g L^−1^ was 5–10 times higher than in the lignocellulosic hydrolysate to account for different kinds of phenolic compounds and lignosulfonates. Defined media were chosen over hydrolysates to have better control over the experiments performed since hydrolysate might contain many unknown inhibitors (Palmqvist and Hahn-Hägerdal 2000) and they might change in composition over time. All media components were sterile filtered to avoid changes in composition due to evaporation.

### Culture conditions

Aerobic cultures were performed at 30 °C in a rotary shake-incubator (New Brunswick, Enfield, CT, USA) at 180 rpm with cell concentrations determined as optical density (OD) at 620 nm (Spectrophotometer U-1800, Hitachi, Berkshire, UK). Seed cultures were grown from single colonies of TMB3500 (YPD agar plate) in 5 mL defined medium in a 50 mL conical tube to reach late exponential phase. Pre-cultures were started from the seed culture in defined medium with an initial OD of 0.5, grown till late exponential phase, unless mentioned otherwise. All the aerobic batch cultures were cultivated in baffled shake flasks with a medium volume equivalent to 10 % of the volume of the baffled shake flask to maintain adequate aeration. Cells for inoculation were obtained after centrifuging the pre-culture at 4000 rpm for 5 min at 4 °C, washing the cells with saline and repeating the centrifugation process. Gas proof neoprene tubes (Masterflex™, Cole-Parmer, Sweden) were used for connections in the anaerobic experiments to avoid oxygen diffusion. All the growth and fermentation experiments except the ALE were carried out at least in biological replicates and measurements were carried out in technical triplicates. Data represented in figures include standard deviations from the replicates.

### Short-term adaptation

Pre-cultures were grown aerobically until late exponential phase in 25 mL of defined medium with the IC at pH 5.0 from the seed culture, termed as short-term adaptation step being used for subsequent cultivations. Cells grown until late exponential phase in defined medium without the IC at pH 5.0 was used as negative control, termed as non-adapted cells. Aerobic batch growth in short-term adaptation experiments were followed for 5 days.

#### Aerobic batch growth

Short-term adapted TMB3500 cells were inoculated into 25 mL of defined media at pH 3.7 with three different inhibitor combinations: (1) 6 g L^−1^ acetic acid and 0.5 g L^−1^ HMF; (2) 6 g L^−1^ acetic acid and 1.5 g L^−1^ furfural; and (3) 6 g L^−1^ acetic acid and 1 g L^−1^ vanillin.

Defined media (25 mL) with the IC at different pH values (pH 5.0, 4.5, 4.0 and 3.7) were inoculated with short-term adapted cells of strain TMB3500 on defined medium.

Short-term adapted TMB3500 cells were inoculated at different cell dry weights (gdw L^−1^) namely 0.5, 1 and 3 gdw L^−1^ into 25 mL of defined medium with the IC at pH 3.7.

#### Anaerobic fermentation

Short-term adapted and non-adapted TMB3500 cells were inoculated at 3 gdw L^−1^ cells into 500 mL of defined medium with the IC at pH 3.7 in a 1 L Infors fermentor (InforsHT, Switzerland). Anaerobic conditions were obtained by sparging with nitrogen gas (200 mL min^−1^), the stirring was set at 200 rpm and the temperature was maintained at 30 °C. The fermentation profile was followed by sampling for metabolites, residual inhibitors and OD.

### Adaptive lab evolution of TMB3500

A pre-culture of strain TMB3500 was used to inoculate an aerobic batch of 1 L defined medium without inhibitors at pH 5.0 in 1.4 L Infors fermentors (InforsHT, Switzerland). The culture was operated at 30 °C, with a stirring rate of 200 rpm and air was sparged at a flow rate of 200 mL min^−1^. At the end of the exponential phase, the feed containing defined medium with the IC was connected to the fermentor at a dilution rate of 0.1 h^−1^ and the fermentor was rendered anaerobic by sparging nitrogen gas at a flow rate of 200 mL min^−1^. After the culture reached steady state, the pH was reduced by 0.2 units using 3 M H_2_SO_4_. A 1.4 L Infors fermentor stirring at 200 rpm and nitrogen gas sparged at 200 mL min^−1^ was used between the fermentor with yeast culture and feed bottle to facilitate a slow transition to the new pH. The culture pH was maintained using 3 M KOH. Once the pH was reduced to 4.5, the dilution rate was increased to 0.15 h^−1^. Gradual reduction in pH was continued until it reached a value of 3.7. The experiment was carried out for 3600 h thereby obtaining 709 generations. Samples were taken for OD and metabolite analysis. Cell dry weight analyses were performed when the culture was in steady state. At the end of the evolution experiment, the cell suspension was transferred to a new chemostat running under the same conditions.

### Characterisation of evolved population

The evolved population (CC156) at pH 3.7 in the ALE and the parental strain TMB3500 were compared for their robustness towards low pH and acetic acid with other inhibitors. In addition, a biofilm was formed when the pH of the chemostat was reduced to 4.1. The biofilm and CC156 population were collected at the end of the chemostat culture and stored as glycerol stocks. They were streaked on YPD plates to obtain single colonies for DNA fingerprinting and used as a population from glycerol stock in the pre-cultures for liquid media growth experiments.

Agar plates were prepared by mixing autoclaved 20 g L^−1^ agar with filter sterilised chemically defined media of different conditions including pH 5.0 and 3.7 (with or without the IC) and pH 4.5 (50 % of the IC). Cell suspensions of strain CC156 and strain TMB3500 (100 μL) were streaked evenly on the agar plates and incubated at 30 °C aerobically and anaerobically for 3–7 days. Experiments were carried out in triplicate to enumerate the colony forming units (CFUs).

Aerobic and anaerobic liquid batch growth experiments were carried out with pre-cultures of strain TMB3500 (from a single colony) and the evolved population CC156. Different conditions were tested in 25 mL defined media, including pH 5.0 and 3.7 (with and without the IC) and pH 4.5 (50 % of the IC). Cells were grown at 30 °C in baffled shake flasks and sealed glass vials with magnetic stirrers and rubber stoppers to follow aerobic and anaerobic growth, respectively.

Cells of CC156 population, biofilm and strain TMB3500 from the short-term adaptation step were inoculated into 25 mL of defined medium with inhibitors at the concentrations similar to the residual inhibitor concentrations present in the CC156 population of the ALE chemostat at pH 3.7 i.e. 0.32 g L^−1^ HMF, 0.46 g L^−1^ furfural, 0.53 g L^−1^ vanillin, 6.0 g L^−1^ acetic acid, and this aerobic batch growth was followed for 2 days.

Cells of strain CC156, biofilm and strain TMB3500 were propagated in 5 mL of YPD liquid medium containing 10 g L^−1^ yeast extract, 20 g L^−1^ peptone and 20 g L^−1^ glucose. Genomic DNA was extracted (Harju et al. 2004) from three colonies in each strain and amplified by PCR (C1000 Touch™ thermocycler, Bio-rad, USA) using Dream taq polymerase (Life technologies, Sweden) and selected primers targeting (1) TY1, TY3 elements (Transposable elements, individually and combined) (i Nogué et al. 2012; Schofield et al. 1995). TY elements were chosen due to their presence in a wide variation in the yeast genome distribution making them ideal for intraspecies discrimination (Schofield et al. 1995), (2) randomly amplified polymorphic DNA (S1254 random primer) (Akopyanz et al. 1992) and (3) (GACA)_4_ (Andrade et al. 2006) and (GTG)_5_ repeats (da Silva-Filho et al. 2005). They were chosen owing to their usefulness in differentiating a strain during the evolution monitoring process in wine fermentation (da Silva-Filho et al. 2005). PCR was performed adopting to conditions from the respective literature. The PCR products were separated in an agarose gel (0.8 %) at 100 V for 60 min with a gene ruler DNA ladder (100 bp–10 kb) and gene ruler 100 bp plus ladder (100 bp–3 kb) (Thermo Scientific, Sweden) as standards.

### Metabolite analysis

Cell dry weight was determined in triplicate by filtering 5 mL of the culture on a pre-weighed 0.45 μm pore size Supor^®^ membrane disc filter (Pall Corporation, Port Washington, NY, USA). Filters were washed with distilled water and dried for 8 min at 350 W in a microwave oven. To analyze the metabolites, cells were separated by centrifugation at 13,200 rpm for 2 min; the supernatant was filtered through 0.20 μm membrane filters (Toyo Roshi Kaish, Tokyo, Japan) and stored at −20 °C until analysis. Concentrations of glucose, glycerol, acetate, ethanol, HMF, furfural and vanillin were determined by high performance liquid chromatography (Waters, Milford, MA, USA) using a HPX-87H resin-based column (Bio-Rad, Hercules, CA, USA) preceded by a Micro-Guard Cation-H guard column (Bio-Rad). Separation was performed at 45 °C with 5 mM H_2_SO_4_ at a flow rate of 0.6 mL min^−1^. All compounds were quantified by refractive index detection (Shimadzu, Kyoto, Japan). For each HPLC run, a seven-point calibration curve was made for each compound.

## Results

In this study, the significance of pre-cultivation and ALE towards low pH and inhibitor tolerance were explored. The TMB3500 yeast strain employed in this study was previously shown to possess high tolerance to lignocellulosic inhibitors by proliferating in the presence of high amounts of non-detoxified hydrolysates, including barley straw (40 % w/v), dilute spruce (60 % w/v) and wheat straw hydrolysates (60 % w/v) (Almeida et al. 2009a).

### Robust phenotype through short-term adaptation and role of individual inhibitors at low pH

**Fig. 1:**
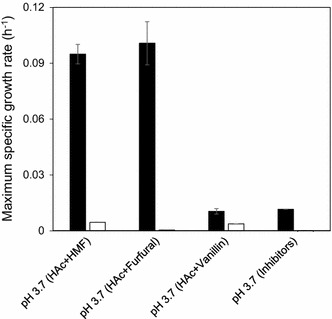
Effect of low pH with 6 g L^−1^ acetic acid and individual inhibitors (1.5 g L^−1^ furfural or 0.5 g L^−1^ HMF or 1 g L^−1^ vanillin) on short-term adapted (*black*) and non-adapted (*white*) cells. pH 3.7 with inhibitors includes the IC

To test the influence of short-term adaptation on tolerance to low pH and individual inhibitors, TMB3500 cells short-term adapted with the IC at pH 5.0 and non-adapted cells were inoculated in defined media at pH 3.7 with acetic acid and individual inhibitors. Short-term adapted TMB3500 was able to grow at pH 3.7 with acetic acid and HMF at a maximum specific growth rate (μ_max_) of 0.09 ± 0.01 h^−1^ without any lag phase (Fig. 1). Cells grown at pH 3.7 with acetic acid and furfural had a μ_max_ of 0.10 ± 0.01 h^−1^ with a lag phase of 13 ± 0.5 h whereas cells grown at pH 3.7 with acetic acid alone had a similar μ_max_ (0.11 ± 0.01 h^−1^), but without any lag phase (Sànchez i Nogué et al. 2013). This indicates that furfural had a combined inhibitory effect along with acetic acid. Short-term adapted cells inoculated at pH 3.7 with acetic acid and vanillin had just marginal growth of 0.01 ± 0.00 h^−1^ after 160 h (Fig. 1). Similarly, minimal growth of 0.01 ± 0.00 h^−1^ was observed in the culture at pH 3.7 with all the inhibitors. Minimal to no growth was observed with non-adapted cells in any of the above conditions, hence subsequent aerobic batch experiments were performed only with short-term adaptation.

### Combined effect of different pH and multiple inhibitors on growth

**Fig. 2:**
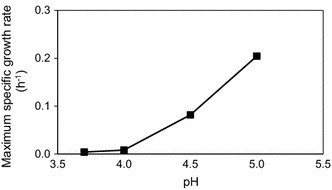
Effect of the pH on growth of strain TMB3500 after short-term adaptation at pH 5 with the IC

To further map the inhibitory effect of low pH and acetic acid in the presence of a cocktail of lignocellulosic inhibitors, a short-term adapted TMB3500 culture was grown at different pH between 3.7 and 5.0 in media with the IC. Whereas the cells at pH 5.0 started growing almost immediately with a μ_max_ of 0.20 ± 0.00 h^−1^, a decrease in pH by 0.5 units resulted in a long lag phase (20 ± 1 h) and a reduction in μ_max_ to 0.08 ± 0.00 h^−1^. At pH 4.0 there was just a marginal growth and no growth was observed at pH 3.7 even after 150 h (Fig. 2). This illustrates the critical role played by pH in the presence of acetic acid and inhibitors on yeast growth.

### Influence of initial cell density in growth performance at low pH with inhibitors

**Fig. 3:**
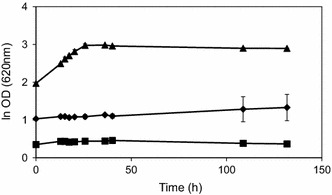
Effect of initial cell density on growth and inhibitor tolerance of strain TMB3500 at pH 3.7 with the IC after short-term adaptation. Cell dry weight-3 gdw L^−1^ (*triangles*), 1 gdw L^−1^ (*diamonds*) and 0.5 gdw L^−1^ (*squares*)

To investigate whether the observed growth inhibition could be relieved by increasing the initial cell density and if there was a critical cell concentration that allowed for growth to occur, short-term adapted TMB3500 cultures were inoculated at different cell concentrations (0.5, 1 and 3 gdw L^−1^) in a medium at pH 3.7 with the IC. When inoculated with 3 gdw L^−1^, cells grew at a μ_max_ of 0.04 ± 0.00 h^−1^ (Fig. 3) in comparison to an inoculum size of 0.5 or 1 gdw L^−1^, where no growth was observed even after 130 h of incubation. The short-term adapted biomass at higher concentration might possess the required volumetric reductase activity to efficiently detoxify HMF and furfural to their corresponding alcohols (Modig et al. 2008) reaching inhibitor threshold levels for allowing growth at the low pH in presence of acetic acid.

### Effect of initial cell density in fermentation performance at low pH with inhibitors

**Fig. 4:**
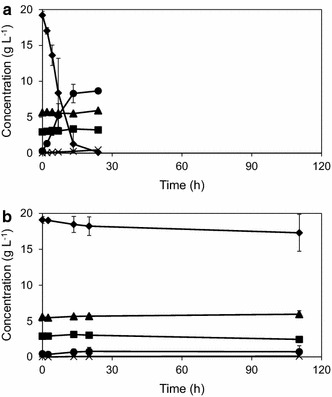
Anaerobic batch fermentation of strain TMB3500 with 3 gdw L^−1^ cells in pH 3.7 with the IC with (**a**) and without (**b**) short-term adaptation. Cell dry weight (*squares*), glucose (*diamonds*), ethanol (*circles*), acetate (*triangles*) and glycerol (*crosses*)

**Fig. 5:**
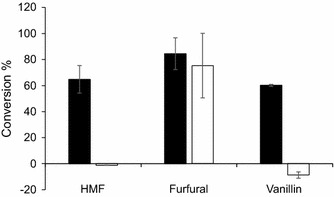
Inhibitor conversion profile in anaerobic batch fermentation with (*black*) and without (*white*) short-term adaptation

To test the influence of the short-term adaptation strategy to maintain metabolic activity and the ability to ferment at pH 3.7 in the presence of IC, an anaerobic batch culture was inoculated with 3 gdw L^−1^ of a TMB3500 culture with (Fig. 4a) and without pre-adaptation (Fig. 4b). In the pre-adapted system, glucose was consumed completely within 19 ± 5 h at a specific consumption rate of 0.47 ± 0.01 g glucose g cells^−1^ h^−1^ to produce 0.45 ± 0.01 g ethanol g glucose^−1^ (Table 1). There was a significant detoxification of inhibitors including 65 ± 10 % of HMF, 85 ± 12 % of furfural and 60 ± 1 % of vanillin to their respective alcohols (Fig. 5). In the non-adapted system, the cell concentration started to decrease and there was no significant sugar consumption or ethanol production even after 110 h and no inhibitor detoxification, except for 75 ± 12 % of furfural (Fig. 5; Table 1).

**Table 1:** Anaerobic batch fermentation at pH 3.7 with the IC with and without short-term adaptation at pH 5

Condition	Ethanol titre (g L^−1^)	Yield (g g glucose^−1^)				Specific consumption/production rate (g g^−1^ h^−1^)	
		Biomass	Glycerol	Acetate	Ethanol	Glucose	Ethanol
Short-term adapted	8.93 ± 0.38	0.01 ± 0.00	0.01 ± 0.01	0.00 ± 0.01	0.45 ± 0.01	−0.47 ± 0.01	0.18 ± 0.02
Non-adapted	0.73 ± 0.84	−0.58 ± 0.56	0.03 ± 0.04	0.30 ± 0.15^a^	0.15 ± 0.13^a^	0.01 ± 0.00	0.00 ± 0.00

^a^Yield values based on glucose consumption of 1.80 ± 2.11 g in total

### ALE of TMB3500 for tolerance towards low pH with acetic acid and other inhibitors

**Fig. 6:**
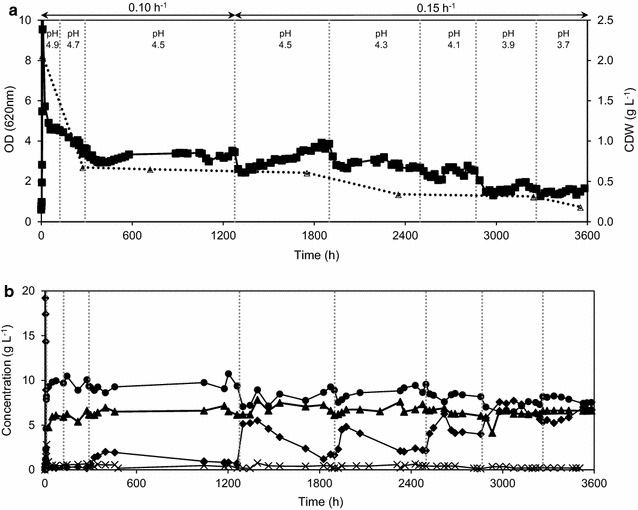
**a** ALE of strain TMB3500. Optical density (*solid squares*) and cell dry weight (*spotted triangles*). *Dotted vertical lines* indicate a change in pH. **b** Metabolite profile of ALE of strain TMB3500. Glucose (*diamonds*), ethanol (*circles*), acetate (*triangles*) and glycerol (*crosses*)

Though short-term adaptation is successful in obtaining a stable growth and fermentation profile at low pH under inhibitory conditions, a stable robust yeast strain that does not require short-term adaptation towards low pH would be more ideal. Cells of strain TMB3500 were evolved to improve their tolerance towards low pH with inhibitory concentrations of acetic acid (6 g L^−1^) and other inhibitors typically present in lignocellulose hydrolysates including furfural (1.5 g L^−1^), HMF (0.5 g L^−1^) and vanillin (1 g L^−1^). They were grown in an anaerobic continuous culture at a dilution rate of 0.1 h^−1^ in a defined medium with the IC at pH 5.0. Once the culture attained steady state, the pH was reduced stepwise from pH 5.0 to 3.7. At pH 4.5, cells were further stressed to grow faster by increasing the dilution rate to 0.15 h^−1^. A biofilm which was formed at pH 4.1 in the chemostat covering the glass walls and baffles increased proportionally during further reduction in pH. At pH 3.7, the cell culture CC156 growing in the presence of lignocellulosic inhibitors was obtained after 709 generations (Fig. 6a, b). The cell suspension of the chemostat was transferred into a new chemostat operating under similar conditions to test its capacity for inhibitor tolerance at pH 3.7 and to identify the role of the biofilm in ALE. The chemostat culture washed out (data not shown) and the CC156 cells stored at −80 °C were chosen as the evolved population.

**Fig. 7:**
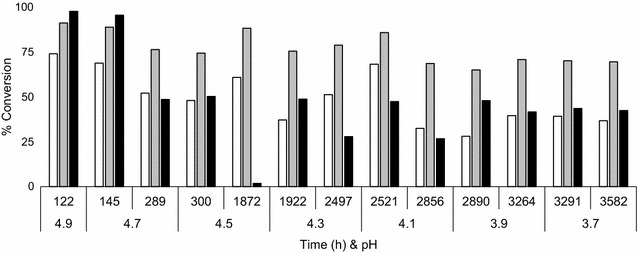
Inhibitor conversion profile throughout the ALE at the start and end of every pH shift. (% conversion, calculated based on initial and measured time point). HMF (*white*), furfural (*grey*) and vanillin (*black*)

The increase in un-dissociated acetic acid concentration in the chemostat due to stepwise lowering the pH led to a series of effects over the course of cultivation:

(1) From pH 5.0 to 4.5 an initial decrease in optical density was followed by recovery of the culture, but at lower pH, the optical density gradually decreased (Fig. 6a); (2) this was accompanied by a temporary increase in the glucose concentration due to a lower glucose rate of consumption. After each pH change, the glucose concentration increased sequentially in the new steady states (Fig. 6b); (3) HMF, furfural and vanillin most probably have been detoxified by NAD(P)H-dependent reductases in strain TMB3500 as observed by Modig et al. (2008) and furfural was detoxified with a conversion efficiency of 75 % on average throughout the chemostat cultivation and HMF and vanillin were being detoxified in varying efficiencies based on the cell concentrations at the given time point (Fig. 7). On the other hand, the ethanol yield was maintained at 0.42 ± 0.04 g ethanol g glucose^−1^ throughout the evolution process in spite of the increase in dilution rate and decrease in pH. Moreover, the concentration of acetate remained constant at 6 g L^−1^, as it could not be consumed as a source of carbon by *S. cerevisiae* under anaerobic conditions (Henningsen et al. 2015) (Fig. 6b).

### Characterisation of long-term evolution

Characterisation of the CC156 population from the chemostat was performed in an attempt to isolate a stable strain displaying robustness towards low pH with inhibitors in comparison to the parental strain, TMB3500. To isolate a strain displaying an inhibitor tolerant phenotype at low pH, cells of CC156 and TMB3500 strains were plated in defined solid media with different concentrations of inhibitors and pH without short-term adaptation. Surprisingly, both the strains displayed similar colony growth characteristics: (1) colonies formed in less inhibitory conditions including defined media at pH 5.0, 3.7 and 4.5 with 50 % of the IC. (2) No colonies were formed in severe inhibitory conditions including pH 5.0 or 3.7 with the IC, indicating that the evolved population could not retain the phenotypic robustness against inhibitors.

To validate the strain robustness, cells from the CC156 population and TMB3500 were grown in chemically defined liquid media with different pH and concentrations of inhibitors, aerobically and anaerobically. However, growth characteristics for both the parental and evolved strain were similar in liquid media as well. Cells were considered to have positive growth characteristics if their OD value had doubled at least threefold. Based on this, there was a difference between the growth patterns in solid and liquid media where growth was displayed in low to medium inhibitory conditions, including chemically defined media at pH 5.0, 3.7 and 4.5 with 50 % of the IC and pH 5.0 with IC. Nevertheless, both parental and adapted strain did not grow in the medium with pH 3.7 and IC like in the severest conditions applied in the chemostat.

Since the evolved strain did not display inhibitor robustness in conditions of the ALE chemostat, it was hypothesised that the thick biofilm generated over the course of the long-term adaptation could have a role in detoxifying the medium and cell proliferation, whereas the free cells present in the suspension also might be growing and fermenting, tolerating the residual inhibitors. To verify this hypothesis and to test whether short-term adaptation helps to retain tolerance in the evolved strain, cells from the biofilm, CC156 and TMB3500 were inoculated in liquid media with inhibitor concentrations similar to the residual inhibitor concentrations in the liquid suspension of the ALE chemostat. Unexpectedly, strain TMB3500 could grow at a maximum specific growth rate of 0.07 ± 0.00 h^−1^ with a lag phase of 12 ± 0.5 h, biofilm cells and the CC156 population did not grow even after 150 h.

To investigate if large rearrangements in the genome had occurred, DNA fingerprinting was carried out with different sets of primers in the cells from the biofilm, population CC156 and strain TMB3500. Interestingly, the gel bands were similar for all analysed strains (Additional file 1: Figure S1) indicating that there were no major genetic re-arrangements in the evolved strain in comparison to the parental strain.

ALE of *S. cerevisiae* TMB3500 strain resulted in a population accompanied by a biofilm displaying robustness at pH 3.7 with the IC coupled with efficient ethanol production. However, when the selection pressure was removed through storage, the parental strain and the evolved population had similar growth characteristics under the various inhibitory conditions. This led to the conclusion that the robustness towards low pH and lignocellulosic inhibitors displayed by the evolved strain from ALE might be a result of population heterogeneity.

## Discussion

Developing inhibitor tolerant *S. cerevisiae* strains fermenting at low pH is very attractive to the cellulosic bioethanol industry owing to challenges including bacterial contamination, reduction in cell viability, longer lag phase due to inhibitor detoxification, formation of undesirable products, lower ethanol yield, productivity and titre (Almeida et al. 2009b; Palmqvist and Hahn-Hägerdal 2000; Skinner and Leathers 2004). In this study, two different adaptation strategies were successfully applied to attain tolerance in *S. cerevisiae* towards more severe conditions than investigated before, i.e. low pH at 3.7 and inhibitory concentrations of acetic acid, furfural, HMF and vanillin.

When a short-term adaptation strategy was applied with the IC, cells were able to grow at pH 5.0 and 4.5, but not at pH 4.0 and 3.7 (Fig. 2). This could be due to synergistic effects of low pH and the different inhibitors, i.e. (1) increased passive diffusion of undissociated acetic acid into the cells leading to acidification of the cytoplasm. Once inside the cell, acetate, being a weak acid, will reduce the intracellular pH. Plasma membrane ATPases and multiple drug resistance transporters pump the protons and acetate anions, respectively, out of the cell by utilising ATP, leading to an energy drain (Caspeta et al. 2015; Piotrowski et al. 2014; Taherzadeh and Karimi 2011), (2) deactivation of key cellular and glycolytic enzymes, membrane and DNA damage caused by furfural and HMF (Piotrowski et al. 2014); (3) vanillin may cause damage to cell membrane integrity (Piotrowski et al. 2014; Trinh Thi My et al. 2014); and (4) reduction of the furans demands excessive reducing power of NAD(P)H that is directed away from ethanol production and anabolism (Almeida et al. 2007, 2009b). All four mechanisms might have contributed to reduced growth capacity, elongated lag phases, lower growth rates and ethanol yields (Almeida et al. 2011; Piotrowski et al. 2014). As the mechanism of detoxification, tolerance, energy and co-factor requirements are different among weak acids, furans and phenolics, the presence of more than one class of inhibitor in the substrate results in an additional burden to the yeast cell.

Short-term adaptation to the inhibitors at pH 5 might have led to a reduction in the cytosolic pH that subsequently has increased the tolerance to acetic acid at pH 4.5, as was earlier observed in another *S. cerevisiae* strain (Fernández-Niño et al. 2015). Hence during incubation at pH 3.7 with the IC, the effect of low pH and diffusion of undissociated acetic acid inside the cell must have been less pronounced. Increasing the cell density combined with short-term adaptation improved the potential of growth and ethanol fermentation capacity at pH 3.7 with the IC in 12 h compared to the non-adapted culture (Figs. 4, 5; Table 1). Higher inoculum concentrations could have several positive effects towards inhibitor detoxification including: (1) ‘Safety in numbers’, i.e. lowering the ratio of inhibitor concentration over cell concentration, which leads to lower detoxification demand per cell; and (2) population heterogeneity, where more representatives of several sub populations could be dedicatedly involved in detoxification of inhibitors, thereby facilitating growth and ethanol production by other sub populations. The heterogeneity could be due to (a) variations in cell growth phase, cycle and cell ageing, and (b) stochasticity in gene expression impacting enzymatic activities leading to variations in metabolic reactions (Avery 2006; Delvigne et al. 2014). Of all the inhibitors, vanillin had a major impact on the growth performance of strain TMB3500 at pH 3.7 in the presence of acetic acid as previously observed by Klinke et al. (2004) affecting the growth of *S. cerevisiae* at a concentration of 0.5 g L^−1^, followed by furfural with an increased lag phase, whereas HMF had no effect (Fig. 1) when compared with cells grown at pH 3.7 with acetic acid (Sànchez i Nogué et al. 2013). Since vanillin is involved in membrane damage, it might aid the cellular entry of acetic acid, thereby affecting the fitness and metabolism rapidly. Synergistic effects of acetic acid and furfural have been reported to negatively affect specific growth rate and ethanol yield (Palmqvist et al. 1999). In addition, effects of the individual and combination of inhibitors including furfural, phenol and acetic acid have been analysed using metabolic profiling. The synergistic negative effect on amino acids and central carbon metabolism was more pronounced than the sum of individual inhibitors with acetic acid playing a key role in the combined inhibition (Ding et al. 2011).

Evolutionary engineering is a useful tool to obtain a desired phenotype by acquiring a stable genotype in an organism by applying constant or increasing selection pressure (Almario et al. 2013; Sauer 2001). The natural evolution process towards desirable properties like adaptability to low pH and inhibitor tolerance is prominent among industrial yeast strains where cells are repeatedly washed with dilute H_2_SO_4_ and recycled in the fermentation process along with pre-cultures as observed with the PE-2 strain used in Brazilian ethanol production plants (Della-Bianca et al. 2014). Exposure of strain TMB3500 to step-wise reduction in pH from 5 to 3.7 over 3600 h in a chemostat led to successful growth and ethanol production in the presence of inhibitory concentrations of acetic acid, furfural, HMF and vanillin over the whole pH range. Interestingly, at pH 4.1, biofilm was formed in the fermentor, possibly to protect the cells against harsher conditions as was observed for *Zymomonas mobilis* on rice bran hydrolysate forming a protective layer around cells (Todhanakasem et al. 2014). Yeast cells form biofilm through cell–cell adhesion in response to stress through triggering the Ras/cAMP/protein kinase A (PKA) and mitogen activated protein kinase pathways and expressing the *FLO* genes (Verstrepen and Klis 2006). Hence, the biofilm might have contributed to the majority of the detoxification and cell proliferation. In addition, cells released from the biofilm into the liquid suspension may have contributed to ethanol production, but without proliferation. Therefore, when the cell suspension was transferred into another chemostat under the same condition, the culture simply washed out immediately, adding credibility to this interpretation.

Surprisingly, characterisation of the adapted strain acquired from the suspension after 709 generations showed that it possessed a similar phenotype as the parental strain TMB3500 in response to the exposure of low pH and the IC. The apparent robust phenotype had disappeared as soon as the adapted strain was exposed again to the harsh conditions as seen by Wright et al. (2011) when adapting for acetic acid tolerance. However, one drawback of the experimental setup of characterisation could have been the low inoculum size of OD 0.5 (OD of 2.5 corresponds to 1 gdw L^−1^ of strain TMB3500) used to start the aerobic and anaerobic liquid batches, which may very well have been below the critical cell mass to initially detoxify the inhibitors at low pH when compared with the size of inoculum in the chemostat (Fig. 6a).

All in all, this study indicated that the phenotype of both the short-term and long-term adaptation, i.e. growth at pH 3.7 with inhibitors, turned out to be similar with no rigorous genetic changes. Copy number variations of specific genes and single nucleotide polymorphisms obtained from 5 months of evolution might be very specific to be visualised in the broad genomic DNA fingerprinting techniques used for the current analysis. Indeed, short-term adaptation at acidic pH might have a positive influence in epigenetic expression of various stress response genes and transcription factors/activators including *YAP1*, *HAA1* (Anneli et al. 2006; Modig et al. 2008; Wright et al. 2011). Moreover, De Melo et al. (2010) used an industrial strain named JP1 to show that an acidic environment affected cell growth and induced general stress response. The cell tolerance to acidic environment may involve down regulation of transcription and protein synthesis due to PKA based glucose signalling (De Melo et al. 2010). Interestingly, a cross-tolerance phenomenon has been observed in *S. cerevisiae*, meaning that tolerance acquired to one stress enhances resistance to other forms of stress (Gibson et al. 2007). For instance, low pH stress due to inorganic and weak organic acids induces the expression of genes involved in tolerance to heat shock, cell wall assembly, trehalose biosynthesis, tolerance to osmotic stress and glycerol production (Kapteyn et al. 2001; Kawahata et al. 2006). Also, the resulting enhancement in population performance that we observed in ALE could be due to polygenic response as observed by Meijnen et al. (2016) in the case of tolerance towards acetic acid, preserving beneficial mutations and by avoiding any undesirable pleiotropic response as in case of any targeted genetic manipulation (Sauer 2001).

The adaptation patterns observed in our study, including the long-term adaptation, could thus be due to a combination of a variety of genetic changes and stochastic switching that are either triggered by the harsh environment (Acar et al. 2008) or are already present in a subpopulation (Delvigne and Goffin 2014; Levy et al. 2012). In the latter case, phenotypic variability or plasticity exhibited in the subpopulation might enhance the survival of the species when confronted with diverse hostile environments (Delvigne and Goffin 2014; Levy et al. 2012). Most likely, in TMB3500 cultures only the subpopulation readily adapted to the inhibitors at low pH were selected for, which is underlined by the need to apply thick inocula to provide a critical mass of this subpopulation.

We have performed a short-term adaptation and an ALE of an industrial *S. cerevisiae* strain to grow and produce ethanol at low pH in the presence of lignocellulosic inhibitors. The next step would be to perform fermentations with yeast and simulated contaminations of bacteria in a medium stressed with inorganic and weak organic acids in the presence of lignocellulosic inhibitors to analyze the effect of low pH fermentations. This might be a key step towards reduction of bacterial contamination in large-scale lignocellulosic ethanol production. Though the evolved strain obtained from long-term evolution did not maintain stable inhibitor robustness, the short-term adaptation strategy to pH 5 and all inhibitors made it possible for the first time to successfully ferment glucose to ethanol at pH 3.7 in the presence of lignocellulosic inhibitors. Inhibitor tolerance and cell growth in yeast is thus achieved through phenotypic plasticity, i.e. a delicate phenotypic balance underlined by expression of genes relating to stress tolerance, growth and fermentation. Understanding the complex genetic regulation behind phenotypic plasticity and stochastic expression in adaptive evolution might enable us to steer the yeast cells to desirable metabolic responses.

## Additional file

10.1186/s13568-016-0234-8 DNA fingerprinting with genomic DNA extracted from parental strain TMB3500 (first column of gel), evolved strain CC156 (middle column of gel) and biofilm (third column of gel). Primers used include a) (GACA)_4_ b) (GTG)_5_ c) S1254 random primer d) TY1 primer e) TY3 primer f) TY1 + TY3 primer. One gel is displayed from each strain as a representative from triplicate analysis.

## Abbreviations

ALE: adaptive lab evolution
SSF: simultaneous saccharification and fermentation
HMF: hydroxymethylfurfural
YPD: yeast peptone dextrose
KOH: potassium hydroxide
H_2_SO_4_: sulphuric acid
IC: inhibitor cocktail
OD: optical density
TMB3500: Brewer’s yeast strain
CC156: evolved population
TY: transposable elements
μ_max_: maximum specific growth rate
PKA: protein kinase A

## References

[CR1] Acar M, Mettetal JT, van Oudenaarden A (2008). Stochastic switching as a survival strategy in fluctuating environments. Nat Genet.

[CR2] Akopyanz N, Bukanov NO, Westblom TU, Kresovich S, Berg DE (1992). DNA diversity among clinical isolates of *Helicobacter pylori* detected by PCR-based RAPD fingerprinting. Nucl Acids Res.

[CR3] Albers E, Johansson E, Franzén CJ, Larsson C (2011). Selective suppression of bacterial contaminants by process conditions during lignocellulose based yeast fermentations. Biotechnol Biofuels.

[CR4] Almario MPP, Reyes LH, Kao KC (2013). Evolutionary engineering of *Saccharomyces cerevisiae* for enhanced tolerance to hydrolysates of lignocellulosic biomass. Biotechnol Bioeng.

[CR5] Almeida JR, Modig T, Petersson A, Hähn-Hägerdal B, Lidén G, Gorwa-Grauslund MF (2007). Increased tolerance and conversion of inhibitors in lignocellulosic hydrolysates by *Saccharomyces cerevisiae*. J Chem Technol Biotechnol.

[CR6] Almeida JRM, Karhumaa K, Bengtsson O, Gorwa-Grauslund M-F (2009). Screening of *Saccharomyces cerevisiae* strains with respect to anaerobic growth in non-detoxified lignocellulose hydrolysate. Bioresour Technol.

[CR8] Almeida JRR, Bertilsson M, Gorwa-Grauslund MF, Gorsich S, Lidén G (2009). Metabolic effects of furaldehydes and impacts on biotechnological processes. Appl Microbiol Biotechnol.

[CR7] Almeida JRM, Runquist D, Sànchez Nogué V, Lidén G, Gorwa-Grauslund MF (2011). Stress-related challenges in pentose fermentation to ethanol by the yeast *Saccharomyces cerevisiae*. Biotechnol J.

[CR9] Alriksson B, Horváth IS, Jönsson LJ (2010). Overexpression of *Saccharomyces cerevisiae* transcription factor and multidrug resistance genes conveys enhanced resistance to lignocellulose-derived fermentation inhibitors. Process Biochem.

[CR10] Andrade M, Rodriguez M, Sánchez B, Aranda E, Córdoba J (2006). DNA typing methods for differentiation of yeasts related to dry-cured meat products. Int J Food Microbiol.

[CR11] Anneli P, João RMA, Tobias M, Kaisa K, Bärbel HH, Marie FGG, Gunnar L (2006). A 5-hydroxymethyl furfural reducing enzyme encoded by the *Saccharomyces cerevisiae* ADH6 gene conveys HMF tolerance. Yeast.

[CR12] Avery SV (2006). Microbial cell individuality and the underlying sources of heterogeneity. Nat Rev Micro.

[CR13] Basso LC, Basso TO, Rocha SN. Ethanol production in Brazil: the industrial process and its impact on yeast fermentation. In: dos Santos Bernardes MA, editor. Biofuel production-recent developments and prospects. Rijeka: InTech; 2011. p. 85–100. doi:10.5772/17047.

[CR14] Beales N (2004). Adaptation of microorganisms to cold temperatures, weak acid preservatives, low ph, and osmotic stress: a review. Compr Rev Food Sci F.

[CR15] Beckner M, Ivey M, Phister T (2011). Microbial contamination of fuel ethanol fermentations. Lett Appl Microbiol.

[CR16] Bischoff KM, Liu S, Leathers TD, Worthington RE, Rich JO (2009). Modeling bacterial contamination of fuel ethanol fermentation. Biotechnol Bioeng.

[CR17] Caspeta L, Castillo T, Nielsen J (2015). Modifying yeast tolerance to inhibitory conditions of ethanol production processes. Front Bioeng Biotechnol.

[CR18] da Silva-Filho EAA, Brito dos Santos SK, Resende AdM, de Morais JOO, de Morais MA, Ardaillon Simões D (2005). Yeast population dynamics of industrial fuel-ethanol fermentation process assessed by PCR-fingerprinting. Antonie Van Leeuwenhoek.

[CR19] De Melo HF, Bonini BM, Thevelein J, Simões DA, Morais MA (2010). Physiological and molecular analysis of the stress response of *Saccharomyces cerevisiae* imposed by strong inorganic acid with implication to industrial fermentations. J Appl Microbiol.

[CR20] Della-Bianca BE, de Hulster E, Pronk JT, van Maris AJA, Gombert AK (2014). Physiology of the fuel ethanol strain *Saccharomyces cerevisiae* PE-2 at low pH indicates a context-dependent performance relevant for industrial applications. FEMS Yeast Res.

[CR21] Delvigne F, Goffin P (2014). Microbial heterogeneity affects bioprocess robustness: dynamic single-cell analysis contributes to understanding of microbial populations. Biotechnol J.

[CR22] Delvigne F, Zune Q, Lara AR, Al-Soud W, Sørensen SJ (2014). Metabolic variability in bioprocessing: implications of microbial phenotypic heterogeneity. Trends Biotechnol.

[CR23] Ding MZ, Wang X, Yang Y, Yuan YJ (2011). Metabolomic study of interactive effects of phenol, furfural, and acetic acid on *Saccharomyces cerevisiae*. OMICS.

[CR24] Dominik H, Uwe S (2008). Identification of furfural as a key toxin in lignocellulosic hydrolysates and evolution of a tolerant yeast strain. Microb Biotechnol.

[CR25] Fernández-Niño M, Marquina M, Swinnen S, Rodríguez-Porrata B, Nevoigt E, Ariño J (2015). The cytosolic pH of individual *Saccharomyces cerevisiae* cells is a key factor in acetic acid tolerance. Appl Environ Microbiol.

[CR26] Gibson BR, Lawrence SJ, Leclaire JPR, Powell CD, Smart KA (2007). Yeast responses to stresses associated with industrial brewery handling. FEMS Microbiol Rev.

[CR27] Graves T, Narendranath NV, Dawson K, Power R (2006). Effect of pH and lactic or acetic acid on ethanol productivity by *Saccharomyces cerevisiae* in corn mash. J Ind Microbiol Biotechnol.

[CR28] Hahn-Hägerdal B, Karhumaa K, Larsson CU, Gorwa-Grauslund M, Görgens J, van Zyl WH (2005). Role of cultivation media in the development of yeast strains for large scale industrial use. Microb Cell Fact.

[CR29] Hanqi G, Jian Z, Jie B (2014). Inhibitor analysis and adaptive evolution of *Saccharomyces cerevisiae* for simultaneous saccharification and ethanol fermentation from industrial waste corncob residues. Bioresour Technol.

[CR30] Harju S, Fedosyuk H, Peterson KR (2004). Rapid isolation of yeast genomic DNA: bust n’Grab. BMC Biotechnol.

[CR31] Henningsen BM, Hon S, Covalla SF, Sonu C, Argyros AD, Barrett TF, Wiswall E, Froehlich AC, Zelle RM (2015). Increasing anaerobic acetate consumption and ethanol yields in *Saccharomyces cerevisiae* with NADPH-specific alcohol dehydrogenase. Appl Environ Microbiol.

[CR32] i Nogué V, Bettiga M, Gorwa-Grauslund MF (2012). Isolation and characterization of a resident tolerant *Saccharomyces cerevisiae* strain from a spent sulfite liquor fermentation plant. AMB Express.

[CR33] Ishola MM, Babapour AB, Gavitar MN, Brandberg T, Taherzadeh MJ (2013). Effect of high solids loading on bacterial contamination in lignocellulosic ethanol production. Bioresources.

[CR34] Jönsson LJ, Alriksson B, Nilvebrant N-O (2013). Bioconversion of lignocellulose: inhibitors and detoxification. Biotechnol Biofuels.

[CR35] Kádár Z, Maltha SF, Szengyel Z, Réczey K, De Laat W (2007). Ethanol fermentation of various pretreated and hydrolyzed substrates at low initial pH. Appl Biochem Biotechnol.

[CR36] Kapteyn J, Ter Riet B, Vink E, Blad S, De Nobel H, Van Den Ende H, Klis F (2001). Low external pH induces HOG1-dependent changes in the organization of the *Saccharomyces cerevisiae* cell wall. Mol Microbiol.

[CR37] Kawahata M, Masaki K, Fujii T, Iefuji H (2006). Yeast genes involved in response to lactic acid and acetic acid: acidic conditions caused by the organic acids in *Saccharomyces cerevisiae* cultures induce expression of intracellular metal metabolism genes regulated by Aft1p. FEMS Yeast Res.

[CR38] Klinke HB, Thomsen AB, Ahring BK (2004). Inhibition of ethanol-producing yeast and bacteria by degradation products produced during pre-treatment of biomass. Appl Microbiol Biotechnol.

[CR39] Koichi T, Yukari I, Jun O, Jun S (2012). Enhancement of acetic acid tolerance in *Saccharomyces cerevisiae* by overexpression of the *HAA1* gene, encoding a transcriptional activator. Appl Environ Microbiol.

[CR40] Lei J, Yu S, Lili X, Bingyin P, Yazhong X, Xiaoming B (2011). Enhanced resistance of *Saccharomyces cerevisiae* to vanillin by expression of *lacA* from *Trametes sp. AH28*-*2*. Bioresour Technol.

[CR41] Levy SF, Ziv N, Siegal ML (2012). Bet hedging in yeast by heterogeneous, age-correlated expression of a stress protectant. PLoS Biol.

[CR42] Limayem A, Ricke SC (2012). Lignocellulosic biomass for bioethanol production: current perspectives, potential issues and future prospects. Prog Energy Combust Sci.

[CR43] Liu ZL (2011). Molecular mechanisms of yeast tolerance and in situ detoxification of lignocellulose hydrolysates. Appl Microbiol Biotechnol.

[CR44] Meijnen J-P, Randazzo P, Foulquié-Moreno MR, van den Brink J, Vandecruys P, Stojiljkovic M, Dumortier F, Zalar P, Boekhout T, Gunde-Cimerman N, Kokošar J, Štajdohar M, Curk T, Petrovič U, Thevelein JM (2016). Polygenic analysis and targeted improvement of the complex trait of high acetic acid tolerance in the yeast *Saccharomyces cerevisiae*. Biotechnol Biofuels.

[CR45] Modig T, Almeida JR, Gorwa-Grauslund MF, Lidén G (2008). Variability of the response of *Saccharomyces cerevisiae* strains to lignocellulose hydrolysate. Biotechnol Bioeng.

[CR46] Muthaiyan A, Limayem A, Ricke SC (2011). Antimicrobial strategies for limiting bacterial contaminants in fuel bioethanol fermentations. Prog Energy Combust Sci.

[CR47] Nielsen F, Tomás-Pejó E, Olsson L, Wallberg O (2015). Short-term adaptation during propagation improves the performance of xylose-fermenting *Saccharomyces cerevisiae* in simultaneous saccharification and co-fermentation. Biotechnol Biofuels.

[CR48] Palmqvist E, Grage H, Meinander NQ, Hahn-Hagerdal B (1999). Main and interaction effects of acetic acid, furfural, and p-hydroxybenzoic acid on growth and ethanol productivity of yeasts. Biotechnol Bioeng.

[CR49] Palmqvist E, Hahn-Hägerdal B (2000). Fermentation of lignocellulosic hydrolysates. II: inhibitors and mechanisms of inhibition. Bioresour Technol.

[CR50] Papoutsakis ET, Pronk JT (2015). Editorial overview: energy biotechnology. Curr Opin Biotechnol.

[CR51] Parawira W, Tekere M (2011). Biotechnological strategies to overcome inhibitors in lignocellulose hydrolysates for ethanol production: review. Crit Rev Biotechnol.

[CR52] Piotrowski JS, Zhang Y, Sato T, Ong I, Keating D, Bates D, Landick R (2014). Death by a thousand cuts: the challenges and diverse landscape of lignocellulosic hydrolysate inhibitors. Front Microbiol.

[CR53] Sànchez i Nogué V, Narayanan V, Gorwa-Grauslund M (2013). Short-term adaptation improves the fermentation performance of *Saccharomyces cerevisiae* in the presence of acetic acid at low pH. Appl Microbiol Biotechnol.

[CR54] Sauer U (2001). Evolutionary engineering of industrially important microbial phenotypes. Advances in biochemical engineering/biotechnology.

[CR55] Schofield MA, Rowe SM, Hammond JR, Molzahn SW, Quain DE (1995). Differentiation of brewery yeast strains by DNA fingerprinting. J Inst Brewing.

[CR56] Serate J, Xie D, Pohlmann E, Donald C, Shabani M, Hinchman L, Higbee A, Mcgee M, La Reau A, Klinger GE, Li S, Myers CL, Boone C, Bates DM, Cavalier D, Eilert D, Oates LG, Sanford G, Sato TK, Dale B, Landick R, Piotrowski J, Ong RG, Zhang Y (2015). Controlling microbial contamination during hydrolysis of AFEX-pretreated corn stover and switchgrass: effects on hydrolysate composition, microbial response and fermentation. Biotechnol Biofuels.

[CR57] Skinner KA, Leathers TD (2004). Bacterial contaminants of fuel ethanol production. J Ind Microbiol Biotechnol.

[CR58] Taherzadeh MJ, Karimi K (2011). Fermentation inhibitors in ethanol processes and different strategies to reduce their effects. Biofuels, alternative feedstocks and conversion processes.

[CR59] Takuya I, Daisuke W, Yoko Y, Koichi T, Jun O, Hiroshi T, Hitoshi S, Jun S (2013). An organic acid-tolerant *HAA1*-overexpression mutant of an industrial bioethanol strain of *Saccharomyces cerevisiae* and its application to the production of bioethanol from sugarcane molasses. AMB Express.

[CR60] Todhanakasem T, Sangsutthiseree A, Areerat K, Young GM, Thanonkeo P (2014). Biofilm production by *Zymomonas mobilis* enhances ethanol production and tolerance to toxic inhibitors from rice bran hydrolysate. New Biotechnol.

[CR61] Tomás-Pejó E, Olsson L (2015). Influence of the propagation strategy for obtaining robust *Saccharomyces cerevisiae* cells that efficiently co-ferment xylose and glucose in lignocellulosic hydrolysates. Microb Biotechnol.

[CR62] Trinh Thi My N, Sakihito K, Shingo I (2014). Importance of glucose-6-phosphate dehydrogenase (G6PDH) for vanillin tolerance in *Saccharomyces cerevisiae*. J Biosci Bioeng.

[CR63] Verduyn C, Postma E, Scheffers WA, Van Dijken JP (1992). Effect of benzoic acid on metabolic fluxes in yeasts: a continuous-culture study on the regulation of respiration and alcoholic fermentation. Yeast.

[CR64] Verstrepen KJ, Klis FM (2006). Flocculation, adhesion and biofilm formation in yeasts. Mol Microbiol.

[CR65] Worley-Morse TO, Deshusses MA, Gunsch CK (2015). Reduction of invasive bacteria in ethanol fermentations using bacteriophages. Biotechnol Bioeng.

[CR66] Wright J, Bellissimi E, de Hulster E, Wagner A, Pronk JT, van Maris AJ (2011). Batch and continuous culture-based selection strategies for acetic acid tolerance in xylose-fermenting *Saccharomyces cerevisiae*. FEMS Yeast Res.

