# Supplementary material for: Adaptation to low pH and lignocellulosic inhibitors resulting in ethanolic fermentation and growth of Saccharomyces cerevisiae
Source: AMB Express. 2016 Aug 26;6(1):59. doi: 10.1186/s13568-016-0234-8 (PMC5001960; doi:10.1186/s13568-016-0234-8)
Supplement: Supplementary file 1 — 10.1186/s13568-016-0234-8 DNA fingerprinting with genomic DNA extracted from parental strain TMB3500 (first column of gel), evolved strain CC156 (middle column of gel) and biofilm (third column of gel). Primers used include a) (GACA)4 b) (GTG)5 c) S1254 random primer d) TY1 primer e) TY3 primer f) TY1 + TY3 primer. One gel is displayed from each strain as a representative from triplicate analysis. [file 13568_2016_234_MOESM1_ESM.pptx]

## Slide 1
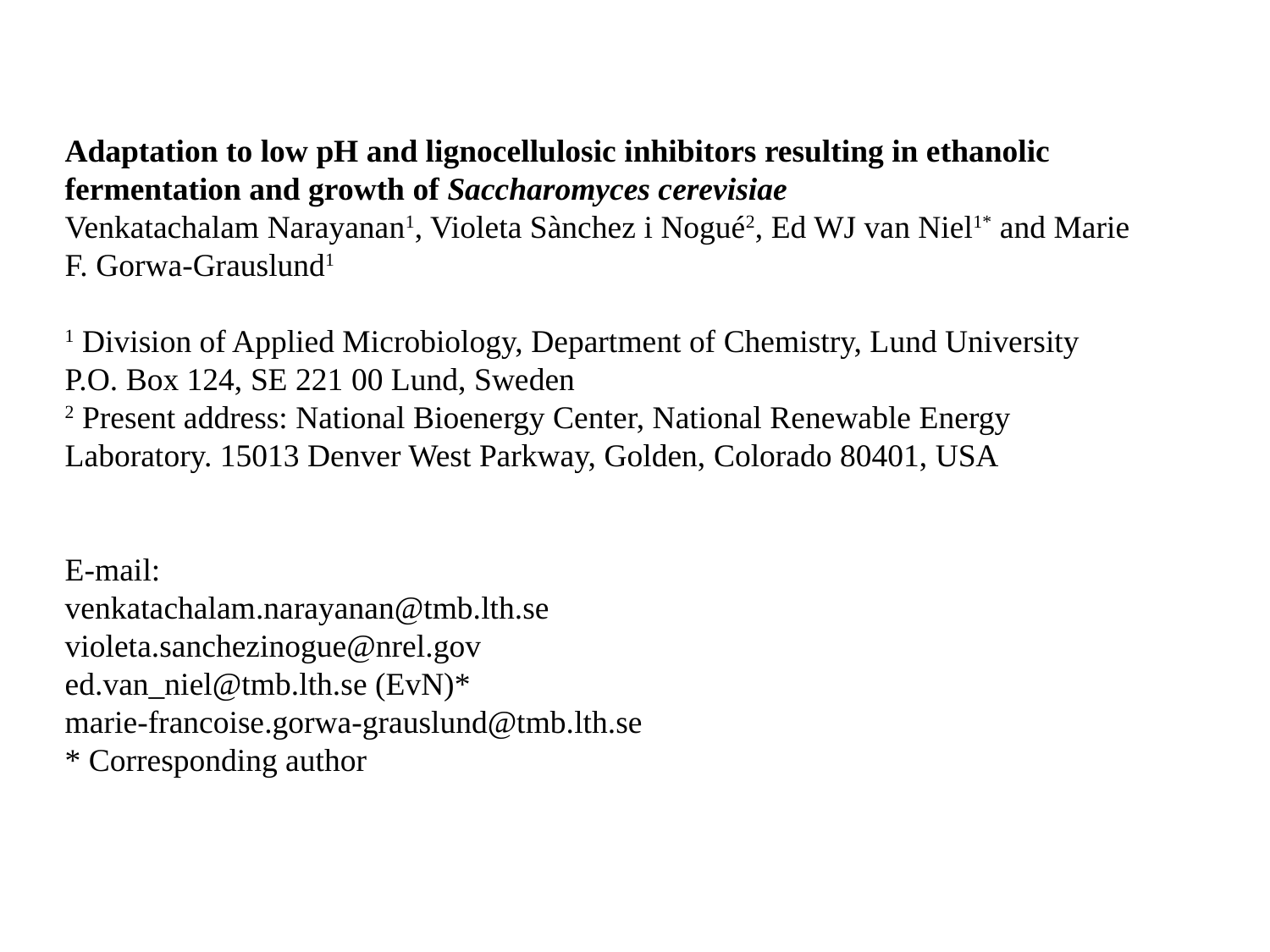

# Adaptation to low pH and lignocellulosic inhibitors resulting in ethanolic fermentation and growth of Saccharomyces cerevisiae Venkatachalam Narayanan1, Violeta Sànchez i Nogué2, Ed WJ van Niel1* and Marie F. Gorwa-Grauslund1   1 Division of Applied Microbiology, Department of Chemistry, Lund University P.O. Box 124, SE 221 00 Lund, Sweden 2 Present address: National Bioenergy Center, National Renewable Energy Laboratory. 15013 Denver West Parkway, Golden, Colorado 80401, USA     E-mail: venkatachalam.narayanan@tmb.lth.se violeta.sanchezinogue@nrel.gov ed.van_niel@tmb.lth.se (EvN)* marie-francoise.gorwa-grauslund@tmb.lth.se * Corresponding author

## Slide 2
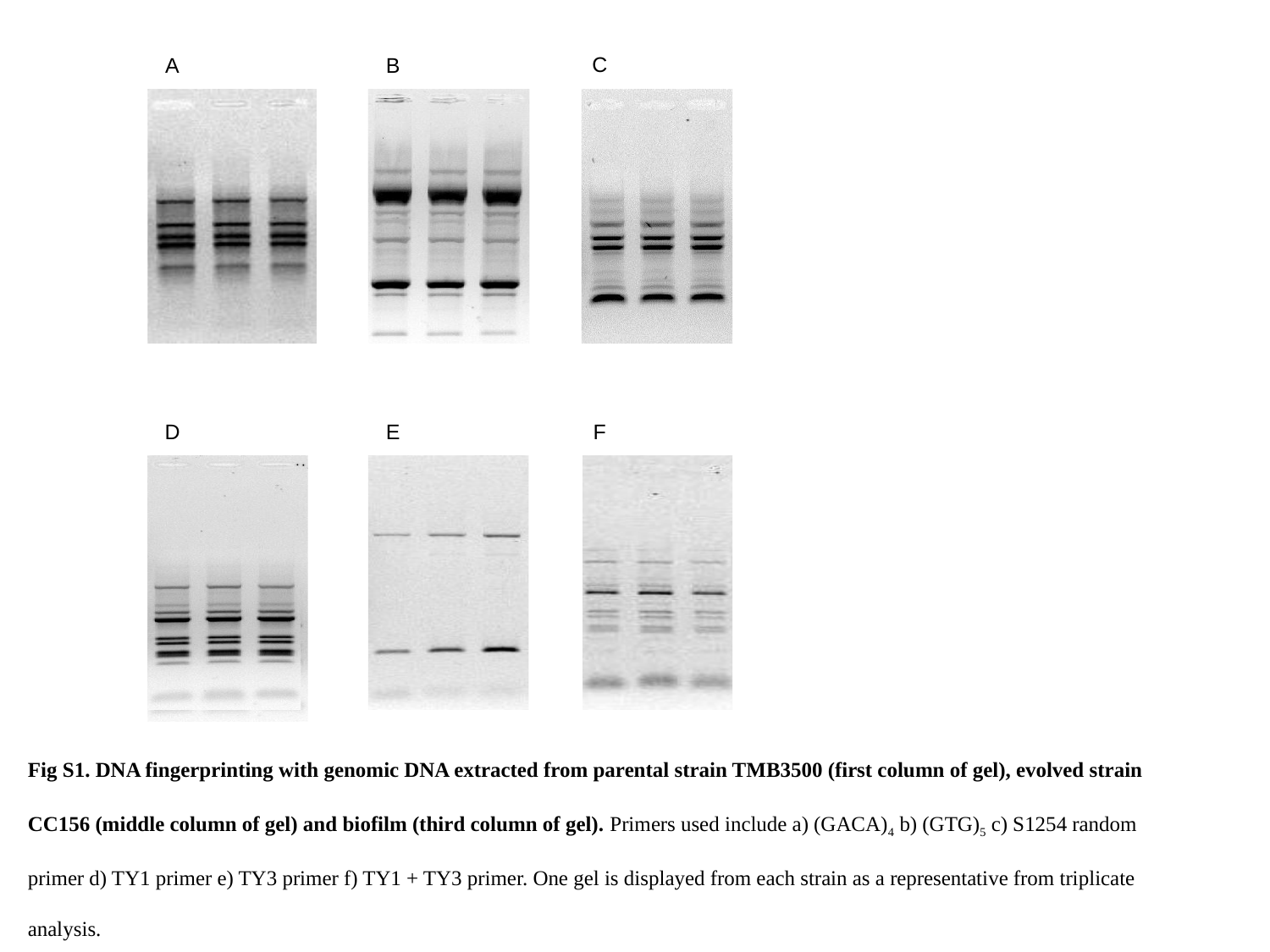

C
B
# A
D
E
F
Fig S1. DNA fingerprinting with genomic DNA extracted from parental strain TMB3500 (first column of gel), evolved strain CC156 (middle column of gel) and biofilm (third column of gel). Primers used include a) (GACA)4 b) (GTG)5 c) S1254 random primer d) TY1 primer e) TY3 primer f) TY1 + TY3 primer. One gel is displayed from each strain as a representative from triplicate analysis.
